# Supplementary material for: Chromothripsis during telomere crisis is independent of NHEJ, and consistent with a replicative origin
Source: Genome Res. 2019 May;29(5):737–49. doi: 10.1101/gr.240705.118 (PMC6499312; doi:10.1101/gr.240705.118)
Supplement: Supplemental Material [file supp_gr.240705.118_Supplemental_file_1.zip › contigs/annotated_contigs/DB51/contig.2.DB51_length_483_mean_cov_4.11801242236.docx]

**DB51_length_483_mean_cov_4.11801242236**

```
TGACTTTAGAAAATAAATATTTTCTTCCTTACTGTACTGAT|TTG|TTGATCTGTCTAATATTTACAGTGGGGAATTGAAGTCTCCCAT
 >chr6:12297391-12297435 + E=7e-12        >chr6:80058630-80059073 - E=1e-247
TATTATTGTGTGGGAGTCTAAGTCTTTTTGTAGGTCTCTAAGGACTTGCTTTATGAACCTGGGTGCTCCTGTATTGGGTGCATATATAT

TTAGGATAGGTAGCTCTTCTTGTTGAATTGATCCCTTTACCATTACGTAGTGGCCTTCTTAGTCTCTTTGGATCTTTGTTGGTTTAAAG

TCTGTTTTATCAGAGACTAGAGTTGCAACCCCTGCTTTTTTTTTTGTTTTCTGTTTGCTTGGTATATCTTCCTCCATCCCTTTATCTTG

AGCCTATGTGTGTCTCTTCACATGAGATGGGTTTCCTGAATACAGCACACTGATGGGTCTTGACTCTTTATCCAATTTGCCAGTCTATG

TCTTTTAATTGGGGTATTTAGCCCATTTACATTTAAGTTA
```
